# Supplementary material for: The interactome of intact mitochondria by cross-linking mass spectrometry provides evidence for coexisting respiratory supercomplexes
Source: Mol Cell Proteomics. 2017 Dec 8;17(2):216–32. doi: 10.1074/mcp.RA117.000470 (PMC5795388; doi:10.1074/mcp.RA117.000470)
Supplement: Supplemental Data [file supp_RA117.000470_133922_0_supp_23978_fzffwf.zip › spectra_annotation/mito_spectra_annotation/164-1-5-1-21-1.pdf]

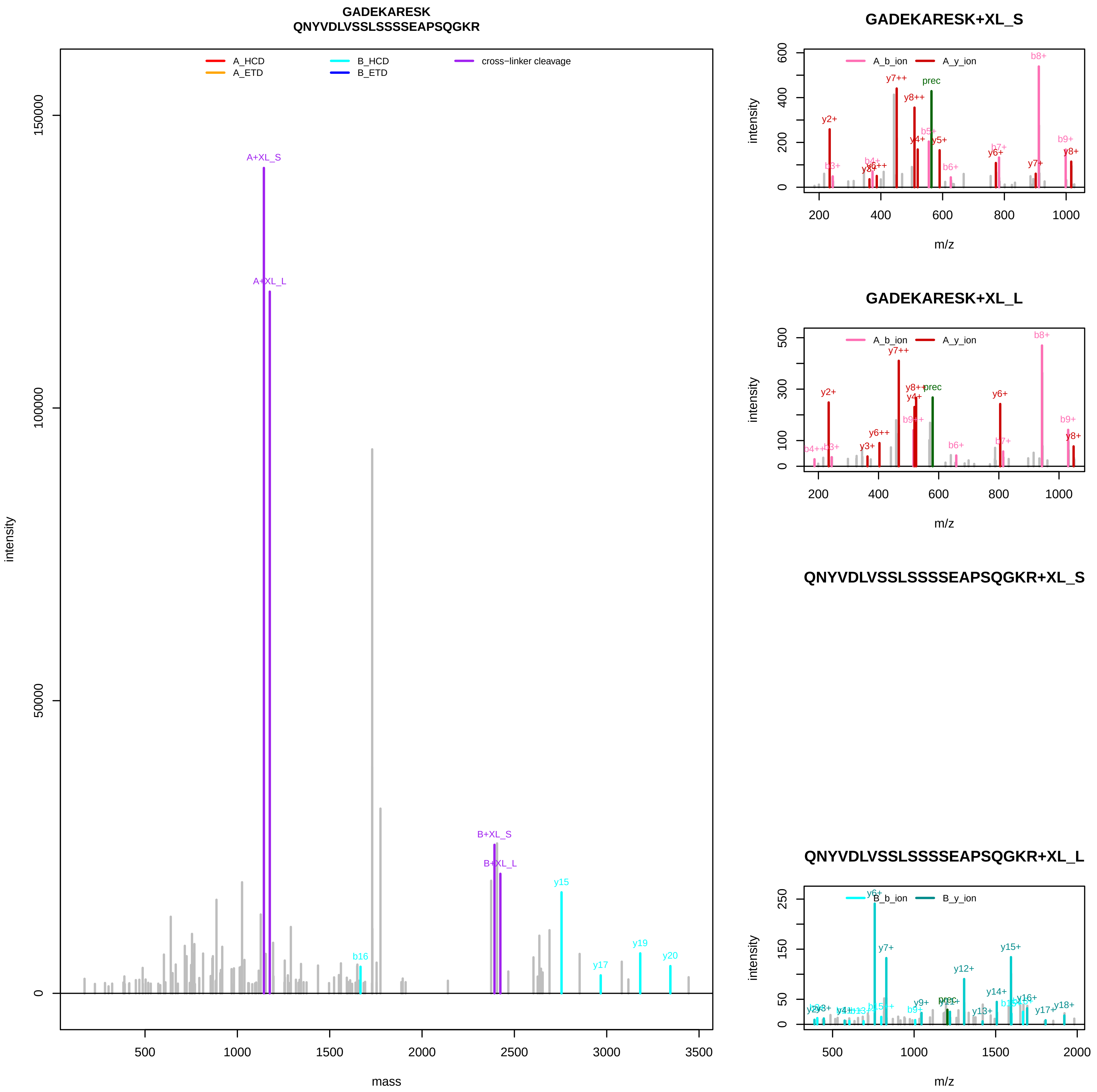
GADEKARESK
QNYVDLVSSLSSSSEAPSQGKR
A_HCD
A_ETD
B_HCD
B_ETD
cross−linker cleavage
intensity
mass
0
50000
100000
150000
500
1000
1500
2000
2500
3000
3500
A+XL_S
A+XL_L
B+XL_S
B+XL_L
b16
y15
y17
y19
y20
GADEKARESK+XL_S
A_b_ion
A_y_ion
m/z
0
200
400
600
800
1000
b8+
y7++
prec
y8++
y2+
b5+
y4+
y5+
b9+
y8+
b7+
y6+
y7+
b3+
b4+
y6++
y3+
b6+
GADEKARESK+XL_L
A_b_ion
A_y_ion
m/z
0
100
200
300
400
500
600
800
1000
b8+
y7++
y2+
y8++
prec
y4+
y6+
b9+
y8+
y6++
y3+
b3+
b4++
b6+
b7+
QNYVDLVSSLSSSSEAPSQGKR+XL_S
QNYVDLVSSLSSSSEAPSQGKR+XL_L
B_b_ion
B_y_ion
m/z
0
50
150
250
500
1000
1500
2000
y6+
y7+
y15+
y12+
y14+
y16+
y9+
prec
y13+
y17+
y18+
y4+
